# Coexistence of *bla*_IMP−4_ and *bla*_SFO−1_ in an IncHI5B plasmid harbored by tigecycline-non-susceptible *Klebsiella variicola* strain

**DOI:** 10.1186/s12941-024-00680-9

**Published:** 2024-03-06

**Authors:** Hui Chen, Hao Xu, Ruishan Liu, Jian Shen, Beiwen Zheng, Lanjuan Li

**Affiliations:** State Key Laboratory for Diagnosis and Treatment of Infectious Diseases, National Clinical Research Center for Infectious Diseases, National Medical Center for Infectious Diseases, Collaborative Innovation Center for Diagnosis and Treatment of Infectious Diseases, The First Affiliated Hospital, Zhejiang University School of Medicine, 79 Qingchun Rd, Hangzhou City, 310003 China

**Keywords:** *Klebsiella variicola*, Tigecycline, IncHI5B, *bla*_IMP−4_, In809

## Abstract

**Background:**

*Klebsiella variicola* is considered a newly emerging human pathogen. Clinical isolates of carbapenemase and broad-spectrum β-lactamase-producing *K. variicola* remain relatively uncommon. A strain of *K. variicola* 4253 was isolated from a clinical sample, and was identified to carry the *bla*_IMP−4_ and *bla*_SFO−1_ genes. This study aims to discern its antibiotic resistance phenotype and genomic characteristics.

**Methods:**

Species identification was conducted using MALDI-TOF/MS. PCR identification confirmed the presence of the *bla*_IMP−4_ and *bla*_SFO−1_ genes. Antibiotic resistance phenotype and genomic characteristics were detected by antimicrobial susceptibility testing and whole-genome sequencing. Plasmid characterization was carried out through S1-PFGE, conjugation experiments, Southern blot, and comparative genomic analysis.

**Results:**

*K. variicola* 4253 belonged to ST347, and demonstrated resistance to broad-spectrum β-lactamase drugs and tigecycline while being insensitive to imipenem and meropenem. The *bla*_IMP−4_ and *bla*_SFO−1_ genes harbored on the plasmid p4253-imp. The replicon type of p4253-imp was identified as IncHI5B, representing a multidrug-resistant plasmid capable of horizontal transfer and mediating the dissemination of drug resistance. The *bla*_IMP−4_ gene was located on the In809-like integrative element (Intl1*-bla*_IMP−4_*-aacA4-catB3*), which circulates in *Acinetobacter* and *Enterobacteriaceae*.

**Conclusions:**

This study reports the presence of a strain of *K. variicola*, which is insensitive to tigecycline, carrying a plasmid harboring *bla*_IMP−4_ and *bla*_SFO−1_. It is highly likely that the strain acquired this plasmid through horizontal transfer. The *bla*_IMP−4_ array (Intl1*-bla*_IMP−4_*-aacA4-catB3*) is also mobile in *Acinetobacter* and *Enterobacteriaceae*. So it is essential to enhance clinical awareness and conduct epidemiological surveillance on multidrug-resistant *K. variicola*, conjugative plasmids carrying *bla*_*IMP−4*_, and the In809 integrative element.

**Supplementary Information:**

The online version contains supplementary material available at 10.1186/s12941-024-00680-9.

## Introduction

The *Klebsiella pneumoniae* complex is a member of the *Klebsiella* genus within the family *Enterobacteriaceae*, including *Klebsiella pneumoniae*, *Klebsiella quasipneumoniae*, and *Klebsiella variicola*. All members of this complex exhibit similar biochemical and phenotypic characteristics, making them indistinguishable using conventional microbiological methods [1, 2]. Furthermore, due to the inability of current microbiological automated detection systems to effectively differentiate *Klebsiella* species, there is a possibility of misidentifying certain isolates of *K. variicola* as *K. pneumoniae* in clinical settings [3–5], leading to an underestimation of the clinical prevalence of *K. variicola. K. variicola* was first described in 2004 and is considered a newly emerging pathogen in humans [6]. It is a gram-negative, facultative anaerobic, non-spore-forming, and non-motile rod-shaped bacterium that forms circular, convex, and smooth colonies [7]. In recent years, with the updating of the MALDI-TOF database and the widespread use of genomic sequencing, there has been an increasing number of reports on isolates of *K. variicola*, including the emergence of highly virulent *K. variicola* strains resistant to colistin [8], the presence of a hypervirulent conjugative plasmid in *K. variicola* [9], and the coexistence of *bla*_NDM−1_ and *bla*_IMP−4_ genes on one plasmid in *K. variicola* [10, 11]. Compared to *K. pneumoniae*, typically, *K. variicola* isolates exhibit lower antibiotic resistance rates [12], but studies have shown a high mortality rate associated with bloodstream infections caused by multidrug-resistant and extended-spectrum β-lactamase-producing *K. variicola* [13, 14]. Therefore, it is crucial to pay close attention to and monitor multidrug-resistant and extended-spectrum β-lactamase-producing *K. variicola*. In this study, the *K. variicola* 4253 strain was isolated from a stool sample of a female leukemia patient in a teaching hospital. The strain was identified to carry the *bla*_IMP−4_ and *bla*_SFO−1_ genes through PCR, and its antibiotic resistance phenotype and genomic characteristics were extensively characterized.

## Materials and methods

### Species identification and antimicrobial susceptibility testing

During routine monitoring targeting at carbapenem-resistant Enterobacteriaceae (CRE) in Hangzhou, Zhejiang Province, China, *K. variicola* 4253 was collected from a stool sample of a female leukemia patient at the First Affiliated Hospital of Zhejiang University. Species identification was conducted using matrix-assisted laser desorption ionization time-of-flight mass spectrometry (MALDI-TOF/MS) (Bruker Daltonik GmbH, Bremen, Germany), while PCR was employed to identify the presence of the carbapenemase gene *bla*_IMP−4_ and the broad-spectrum β-lactamase gene *bla*_SFO−1_ (The primer sequences were showed in Table S1).

Agar dilution method and microbroth dilution method were utilized for antimicrobial susceptibility testing (AST), with *Escherichia coli* ATCC25922 and *Pseudomonas aeruginosa* ATCC27853 serving as the quality control strain. AST results were interpreted according to the guidelines provided by the Clinical and Laboratory Standards Institute (CLSI 2020) and the European Committee on Antimicrobial Susceptibility Testing (EUCAST 2020).

### Plasmid characterization and conjugation experiment

The number and size of plasmids in *K. variicola* 4253 were assessed using S1-PFGE. The location of *bla*_IMP−4_ and *bla*_SFO−1_ on the plasmid was determined through Southern blotting and gene hybridization with a digoxigenin-labeled specific probe (The probe sequences were showed in Table S1). To investigate the transferability of the plasmid, *E. coli* J53 was employed as the recipient strain in conjugation experiments. Transconjugants were selected using Mueller-Hinton agar (OXOID, Hampshire, United Kingdom) supplemented with 200 mg/L NaN3 and 8 mg/L cefepime. Their identification as *E. coli* and the presence of the *bla*_IMP−4_ and *bla*_SFO−1_ genes were confirmed through PCR and further validated using MALDI-TOF/MS.

### Whole genome sequencing and analysis

Genomic DNA was extracted using the Genomic DNA Isolation Kit (QIAGEN, Hilden, Germany), and sequencing was performed using the Illumina Novaseq6000 platform (Illumina, San Diego, CA, USA) and the Oxford Nanopore platforms (Oxford Nanopore Technologies, Oxford, United Kingdom). Following sequencing, the short and long reads were subjected to hybrid assembly using Unicycler v0.4.7 to obtain the complete genome sequence. Annotation was carried out using the RAST 2.0 and Prokka tools. Kleborate v2.0.1 and Kaptive v0.7.3 were utilized for subspecies, MLST, wzi allele, capsule (K), and O antigen (LPS) serotype analysis. Resistance genes were identified using Resfinder. Virulence genes were obtained from the VFDB website, while plasmid replicon types were determined using PlasmidFinder 2.1 and KpVR. Integrons and insertion sequence elements were disclosed using INTEGRALL and ISfinder, respectively. Comparative analysis of different plasmid genome sequences was performed using the BLAST Ring Image Generator (BRIG). Then, the genetic context encompassing the *bla*_IMP−4_ and *bla*_SFO−1_ genes was visualized using Easyfig 2.3.

## Results

### Isolation of *K. Variicola* 4253 strain and antimicrobial susceptibility testing

The *K. variicola* 4253 was recovered from a fecal sample of a 22-year-old female patient with acute lymphocytic leukemia on November 8th, 2021. During hospitalization, the patient experienced high fever and received intravenous administration of cefoperazone sodium, sulbactam sodium, and vancomycin for treatment. After improvement of symptoms, the patient was discharged. The strain was identified as *K. variicola* using MALDI-TOF/MS, and further characterization through third-generation sequencing, using the Kleborate tool, confirmed it as *K. variicola* subsp. *variicola*. PCR and sequencing revealed the presence of the *bla*_IMP−4_ and *bla*_SFO−1_ genes.

As shown in Table 1, the antimicrobial susceptibility results demonstrated that both the strain and its transmissible conjugate exhibited resistance to broad-spectrum β-lactam drugs, including ceftriaxone, cefotaxime, ceftazidime, cefepime, aztreonam, piperacillin/tazobactam, and amoxicillin-clavulanic acid. However, they were susceptible to meropenem and imipenem. They also showed sensitivity to quinolones (levofloxacin, ciprofloxacin), sulfonamides (trimethoprim/sulfamethoxazole), and aminoglycosides (except for gentamicin, which showed resistance but amikacin showed sensitivity). Both the strain and its conjugate exhibited sensitivity to colistin. *K. variicola* 4253 demonstrated resistance to fosfomycin and tigecycline, while its conjugate remained susceptible to them.

**Table 1:** MIC values of antimicrobials for *K. variicola* 4253, transconjugant 4253-J53, and recipient strain J53

	MIC values(mg/L)		
Antimicrobials	*K. variicola* 4253	4253-J53	J53
Ceftriaxone	> 128(R)	128(R)	0.06(S)
Cefotaxime	> 128(R)	128(R)	0.125(S)
Ceftazidime	> 128(R)	> 128(R)	0.5(S)
Cefepime	64(R)	32(R)	0.06(S)
Aztreonam	> 128(R)	64(R)	0.25(S)
Piperacillin/Tazobactam^a^	> 128/4(R)	128/4(R)	4/1(S)
Amoxicillin-Clavulanic acid	> 128/64(R)	128/64(R)	4/2(S)
Imipenem	1(S)	1(S)	0.5(S)
Meropenem	1(S)	0.5(S)	0.03(S)
Fosfomycin	> 512(R)	2(S)	1(S)
Levofloxacin	0.25(S)	0.125(S)	0.06(S)
Ciprofloxacin	0.125(S)	0.008(S)	0.004(S)
Amikacin	4(S)	2(S)	2(S)
Gentamicin	128(R)	64(R)	1(S)
Trimethoprim/Sulfamethoxazole	0.25/4.75(S)	0.125/2.375(S)	0.125/0.357(S)
Tigecycline	8(R)	1(S)	0.5(S)
Colistin	0.5(S)	0.125(S)	0.125(S)

R: resistant; S: susceptible

^a^Tazobactam at a fixed concentration of 4 mg/L

### Genomic features of the strain

**Fig. 1:**
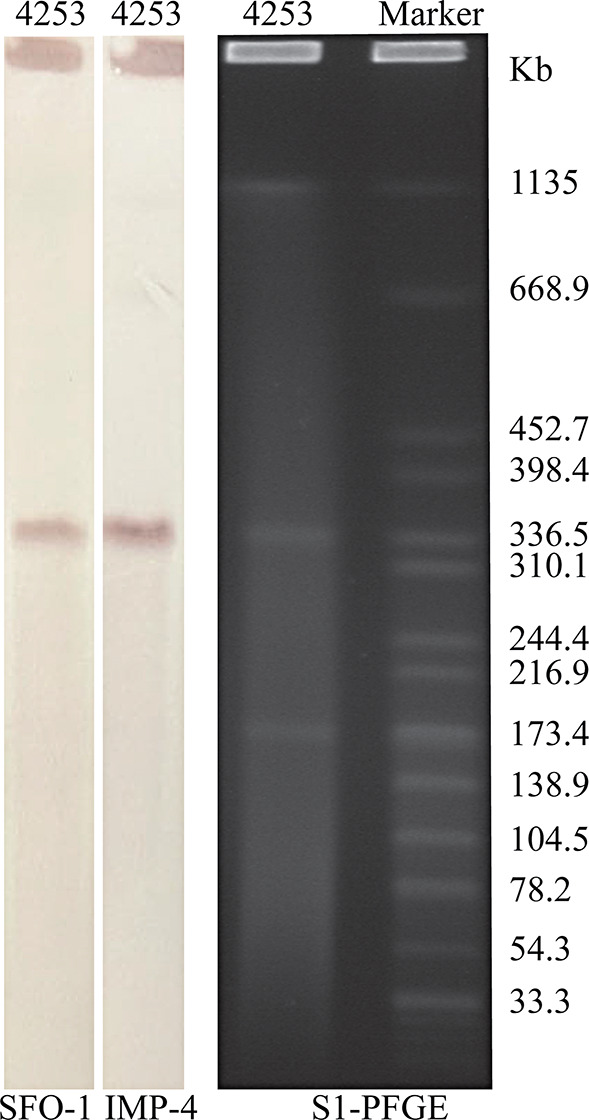
Plasmid profiles of *K. variicola* 4253. Plasmid size determination by S1-PFGE, with *Salmonella enterica* serotype Braenderup H9812 as the size marker. Southern blotting hybridization with the *bla*_SFO−1_-specific probe and *bla*_IMP−4_-specific probe

Genomic characteristics of *K. variicola* 4253 were explored through whole-genome sequencing. The genomic sequence analysis revealed that the isolated strain belongs to ST347 (*gapA-infB-mdh-pgi-phoE-rpoB-tonB*, allele no. 16-24-21-27-47-22-67). The capsule serotype was identified as *wzi*269/KL25/K25, and the O antigen serotype was classified as OL5/O5. The strain possessed a circular chromosome with a size of 5,533,843 bp and three plasmids, namely p4253-imp, p4253-2, and p4253-3. The sizes of the plasmids were 334,271 bp, 175,117 bp, and 4,655 bp, respectively (p4253-3 is not visible in S1-PFGE due to its small size) (Fig. 1). The average G + C content of the chromosome and plasmids were 57.3%, 48.7%, 51.5%, and 42.8%, respectively. Notably, the *bla*_IMP−4_ and *bla*_SFO−1_ genes were located on the same plasmid (p4253-imp). There were 2,651 protein-coding genes, 171 tRNA genes, and 61 rRNA genes on the chromosome. (Table S2).

A total of nine different antimicrobial resistance genes were identified in the strain, including beta-lactamase genes (*bla*_OXA−1_, *bla*_IMP−4_, *bla*_SFO−1_, *bla*_TEM−214_, *bla*_LEN−25_), aminoglycoside resistance genes (*aac* [3]*-IId*, *aac(6’)-Ib-cr*, *aac(6’)-Ib4*, *aph* [6]*-Id*, and *aph(3’’)-Ib*), macrolide resistance genes (*mphA*, *mphE*, and *msrE*), phenicol resistance gene (*catB3*), rifampicin resistance gene (*arr-3*), sulfonamide resistance gene (*sul1*), tetracycline resistance gene (*tet(D)*), fosfomycin resistance gene (*fosA*), and efflux pump-associated genes (*oqxA and oqxB*), of which seven were encoded on plasmid p4253-imp (Table 2), indicating that p4253-imp is a multidrug-resistant plasmid.

**Table 2:** Antibiotic resistance genes of *K.variicola* 4253

Genome	Drug class	Coding genes
Chromosome	Efflux pump-associated gene	*oqxA, oqxB*
	Beta-lactam	*bla*_LEN−25_
	Fosfomycin	*fosA*
p4253-imp	Beta-lactam	*bla*_IMP−4_, *bla*_SFO−1_, *bla*_OXA−1_, *bla*_TEM−214_
	Aminoglycoside	*aac* [3]*-IId, aac(6’)-Ib-cr, aac(6’)-Ib4, aph* [6]*-Id, aph(3’’)-Ib*
	Macrolide	*mphA, mphE, msrE*
	Phenicol	*catB3*
	Rifampicin	*arr-3*
	Sulfonamide	*sul1*
	Tetracycline	*tet(D)*
p4253-2	None	None
p4253-3	None	None

Furthermore, a total of 56 virulence genes were identified in the strain (Table S3). The chromosome harbored genes such as type 3 fimbriae (*mrkABCDFHIJ*), type 1 fimbriae (*fimABCDEFGHIK*), fimbrial adherence determinants (*stcB*, *stcC*), efflux pump (*acrA*, *acrB*), aerobactin (*iutA*), enterobactin siderophore (*entABCDEFS*, *fepABCDG*, *fes*), salmochelin (*iroE*), magnesium uptake (*mgtB*), regulation (*rcsA*, *rcsB*), T6SS-I (*tssJGFM*), T6SS-II (*clpV*), T6SS-III (*dotU*, *icmF*, *impJGHFA*, *ompA*, *sciN*, *vgrG*). The plasmid p4253-2 carried the T6SS-I gene (*tssH*), while no virulence genes were found on other plasmids.

### Characterization of the p4253-imp plasmid

**Fig. 2:**
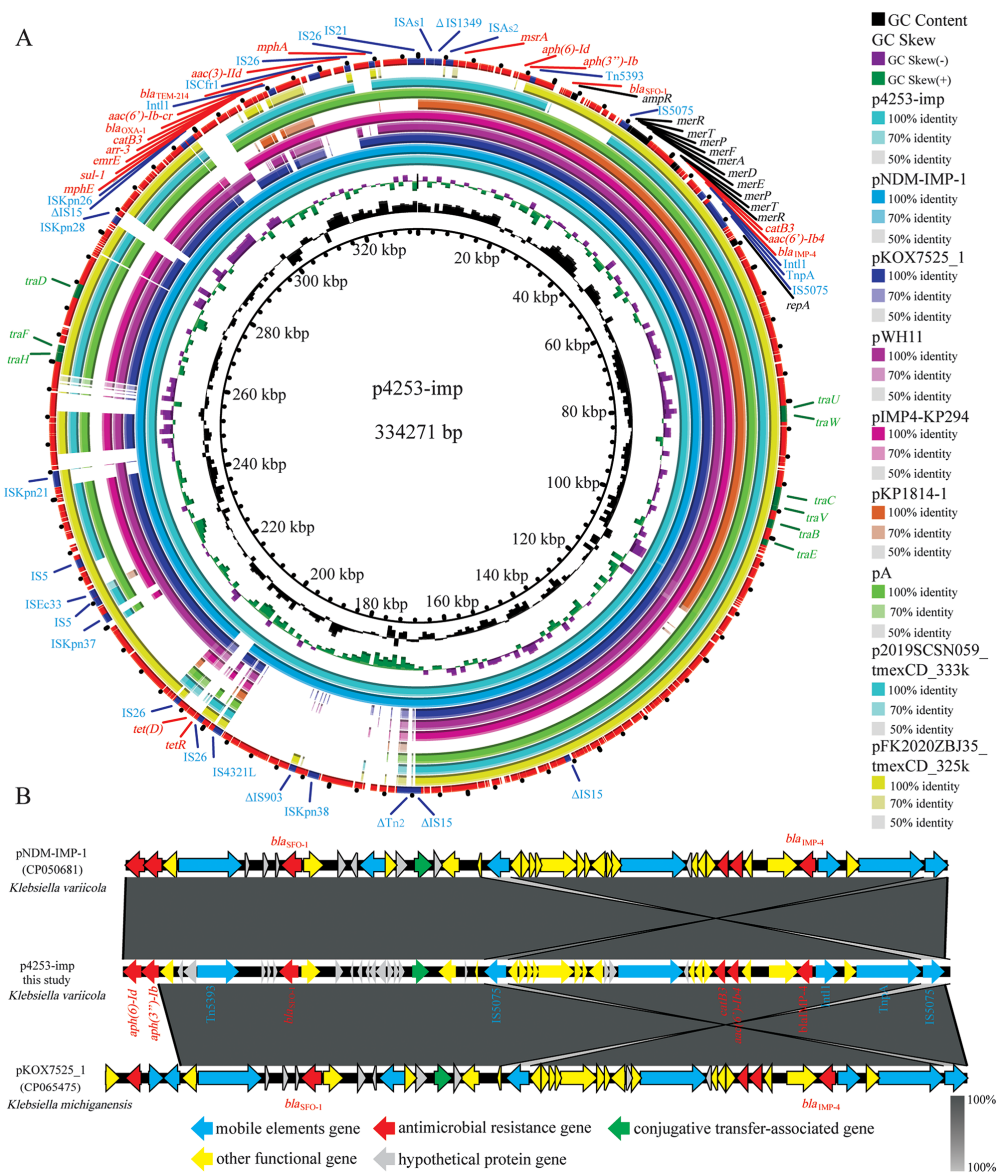
The genomic analysis of *K. variicola* 4253. (**A**) Comparative analysis of plasmid p4253-imp with other eight plasmids.. (**B**) The genetic context of *bla*_SFO−1_ and *bla*_IMP−4_ genes on p4253-imp, pNDM-IMP-1 and pKox7525_1

The size of the p4253-imp plasmid was 334,271 bp. It encoded 318 protein-coding genes and had a G + C content of 48.7%. No hits were found in the Plasmidfinder database for plasmid typing; however, analysis using the KpVR online tool revealed that the p4253-imp replicon belonged to the IncHI5B subtype. Based on this information, it can be inferred that the plasmid is a multidrug-resistant plasmid, carrying carbapenemase gene *bla*_IMP−4_ and broad-spectrum β-lactamase gene *bla*_SFO−1_. Consistent with the genomic features, S1-PFGE and Southern blot confirmed the size of the plasmid and the presence of *bla*_IMP−4_ and *bla*_SFO−1_ on the p4253-imp plasmid (Fig. 1). BLASTN search showed a high similarity between p4253-imp and pNDM-IMP-1 (CP050681.1), pKOX7525_1 (CP065475.1), pWH11 (ON882017.1), pIMP4-KP294 (CP083446.1), pKP1814-1 (KX839207.1), pA (CP068445.1), p2019SCSN059_tmexCD (ON169978.1), and pFK2020ZBJ35_tmexCD (ON169979.1) (More details about the 8 plasmids are showed in Table S4), with identity and coverage both exceeding 90%. Among these, p4253-imp exhibited higher similarity to pNDM-IMP-1, with 99% identity and 100% query coverage. pNDM-IMP-1 is a plasmid carrying *bla*_IMP−4_ from a urine catheter isolate of *K. variicola* SHET-01, obtained from a 2-year-old girl in the pediatric intensive care unit of a teaching hospital in Shanghai, China [11]. Gene comparison analysis (Fig. 2) revealed that p4253-imp and pNDM-IMP-1 share a similar plasmid backbone, including resistance determinants, insertion elements, conjugative transfer gene clusters, and related functional genes. Similar to the pNDM-IMP-1 plasmid, *bla*_IMP−4_ was carried by a Tn6406-like composite transposon, flanked by IS5075 elements, and containing an In809-like integron (Intl1*-bla*_IMP−4_*-aacA4-catB3*). The upstream sequence was identical to Tn5393-*bla*_SFO−1_-*ampR*-IS5075, where Tn5393 harbored *aph* [6]*-Id* and *aph(3’’)-Ib* resistance genes. Furthermore, conjugation experiments demonstrated the transferability of p4253-imp, and the antimicrobial susceptibility results indicated that this plasmid mediated the spread of resistance.

## Discussion

The antimicrobial susceptibility test indicated that *K. variicola* 4253 exhibited.

insensitivity to tigecycline (MIC 8 mg/L). Through whole-genome analysis, no plasmid-mediated *tet(X)* gene encoding tetracycline inactivation enzyme or resistance-nodulation-division (RND) genes conferring tigecycline resistance were identified [15, 16]. Additionally, no conjugate conferring tigecycline insensitivity were obtained in the conjugation experiment. Therefore, it is postulated that the insensitivity of this strain to tigecycline is mediated by chromosomally carried genes. Previously reported mechanisms of tigecycline resistance in *K. pneumoniae* included mutations in the ribosomal protein S10 encoding gene *rpsJ*, which mediates tigecycline resistance [17], the involvement of the efflux pump OqxAB in low to moderate levels of tigecycline resistance [18], overexpression of the AcrAB efflux pump affecting sensitivity to tigecycline in *K. pneumoniae* [19–21], and mutations, insertions, deletions, and upregulation of transcriptional regulatory factors and related genes such as *rarA*, *acrR*, *ramA*, *ramR*, *marA*, and *soxS*, which can regulate the expression levels of the OqxAB and AcrAB efflux pumps and influence sensitivity to tigecycline [22–24]. In this study, the chromosomal genome of the strain contained the efflux pump genes *acrAB-tolC* and *oqxAB*. However, whether the tigecycline-insensitive phenotype in this strain is caused by overexpression of the efflux pump or mutations in related genes remains to be determined and requires further experimentation.

Although the isolated strain *K. variicola* 4253 produces carbapenemase, the antimicrobial susceptibility test revealed its sensitivity to imipenem and meropenem (MIC 1 mg/L). Previous literature reports have shown heterogeneity in the sensitivity of *Enterobacteriaceae* isolates carrying *bla*_IMP−4_ gene to carbapenems. For instance, Yang et al. reported that most of the 25 IMP-producing *K. pneumoniae* (IMPKp) isolates (including 16 IMP-4 strains) collected from a teaching hospital in China between 2009 and 2016 exhibited low levels of resistance and susceptibility to various carbapenem drugs [25]. Among them, three strains demonstrated intermediate to high-level resistance (MIC ≥ 8 mg/L) to imipenem and meropenem. Qiao et al. reported a *Enterobacter hormaechei* strain carrying *bla*_IMP−4_, which showed intermediate susceptibility to imipenem and sensitivity to meropenem [26]. Additionally, *K. pneumoniae* 1220, isolated from a three-month-old infant’s blood specimen and carrying the *bla*_IMP−4_ gene on a plasmid, was capable of conjugation into *E. coli* EC600. The antimicrobial susceptibility results indicated that both the strain and its conjugative form exhibited resistance to imipenem and meropenem (MIC 8 mg/L) [27]. It is speculated that IMP-4 hydrolyzing enzymes may have weak hydrolytic capabilities against carbapenems, and the production of IMP-4 is not the sole determinant for high-level carbapenem resistance. Other resistance mechanisms, including overexpression of efflux systems, porin protein deficiencies, and the presence of other broad-spectrum β-lactamases, may contribute to this process [28]. Currently, the extent to which different resistance mechanisms impact the carbapenem-resistant phenotype in *Enterobacteriaceae* strains carrying *bla*_IMP−4_ remains unclear.

The genetic context of *bla*_IMP−4_ in *K. variicola* 4253 was represented by Intl1*-bla*_IMP−4_*-aacA4-catB3*, which is similar to the integrative element In809 (Intl1*-bla*_IMP−4_*-qacG-aacA4-catB3*). It is possible that the *qacG* gene cassette was lost during the dissemination process. In809 was first described in *Acinetobacter* species isolated from Hong Kong between 1997 and 2000 [29] and has been subsequently reported in various *Acinetobacter* [30]and *Enterobacteriaceae* isolates [11, 25, 31]. This suggests that the *bla*_IMP−4_ array may be mobilized in *Acinetobacter* and *Enterobacteriaceae* through homologous recombination or plasmid-mediated horizontal transfer. Plasmid-mediated dissemination of carbapenemase-encoding genes plays a significant role in the emergence and spread of carbapenem-resistant *Enterobacteriaceae* (CRE). Previously reported incompatible plasmid types carrying the *bla*_IMP_ gene include HI2/HI5, FI/FII, L/M, A/C, P, and N [32, 33]. However, in this study, the plasmid carrying *bla*_IMP−4_, p4253-imp, belonged to the IncHI5B type. IncHI plasmids are conjugative plasmids typically larger than 200 kb, serving as important vehicles for the dissemination of heavy metal resistance genes and antibiotic resistance genes. They are considered broad-host-range plasmids [34]. The conjugation experiments in this study also confirmed that p4253-imp was a conjugative plasmid capable of mediating horizontal transfer of resistance genes. BLAST search revealed an extremely high similarity between p4253-imp and the plasmid pNDM-IMP-1 isolated from *K. variicola* SHET-01 in Shanghai [11]. Given the geographic proximity between Shanghai and Hangzhou, it raises the possibility of clonal dissemination. Based on multilocus sequence typing (MLST), *K. variicola* SHET-01 belonged to ST3936, while *K. variicola* 4253 in this study belonged to ST347. Therefore, it is inferred that the strain isolated in this study is more likely to have been acquired through plasmid-mediated horizontal transfer.

## Conclusion

We have reported the presence of a strain of *K. variicola*, which was insensitive to tigecycline, carrying a plasmid harboring *bla*_IMP−4_ and *bla*_SFO−1_. We have provided a detailed exposition of its drug resistance phenotype and genomic characteristics. This strain was classified as a multidrug-resistant strain, and its one plasmid coexisting *bla*_IMP−4_ and *bla*_SFO−1_, possessed the ability of horizontal transfer, thereby mediating the dissemination of drug resistance. Additionally, it is highly probable that *K. variicola* 4253 acquired this plasmid through plasmid-mediated horizontal transfer. Furthermore, the *bla*_IMP−4_ array (Intl1*-bla*_IMP−4_*-aacA4-catB3*) is also mobile in *Acinetobacter* and *Enterobacteriaceae*. Thus, it is imperative to enhance clinical awareness and conduct epidemiological surveillance in this regard.

### Electronic supplementary material

Below is the link to the electronic supplementary material.

Supplementary Material 1

Supplementary Material 2

## Data Availability

The complete sequence of *K. variicola* 4253 has been submitted to GenBank under accession no. CP135068-CP135071.

## Abbreviations

S1-PFGE: S1-nuclease pulsed-filed gel electrophoresis
PCR: Polymerase chain reaction
MALDI-TOF/MS: Matrix-assisted laser desorption ionization time-of-flight mass
MLST: Multilocus sequence typing
CRE: Carbapenem-resistant Enterobacteriaceae
AST: Antimicrobial susceptibility testing
WGS: Whole-genome sequencing
CLSI: Clinical and Laboratory Standards Institute
EUCAST: European Committee on Antimicrobial Susceptibility Testing
MIC: Minimum inhibitory concentration
