# Supplementary material for: Coexistence of blaIMP−4 and blaSFO−1 in an IncHI5B plasmid harbored by tigecycline-non-susceptible Klebsiella variicola strain
Source: Ann Clin Microbiol Antimicrob. 2024 Mar 6;23:24. doi: 10.1186/s12941-024-00680-9 (PMC10918965; doi:10.1186/s12941-024-00680-9)

1. The original, uncropped, full-length gel


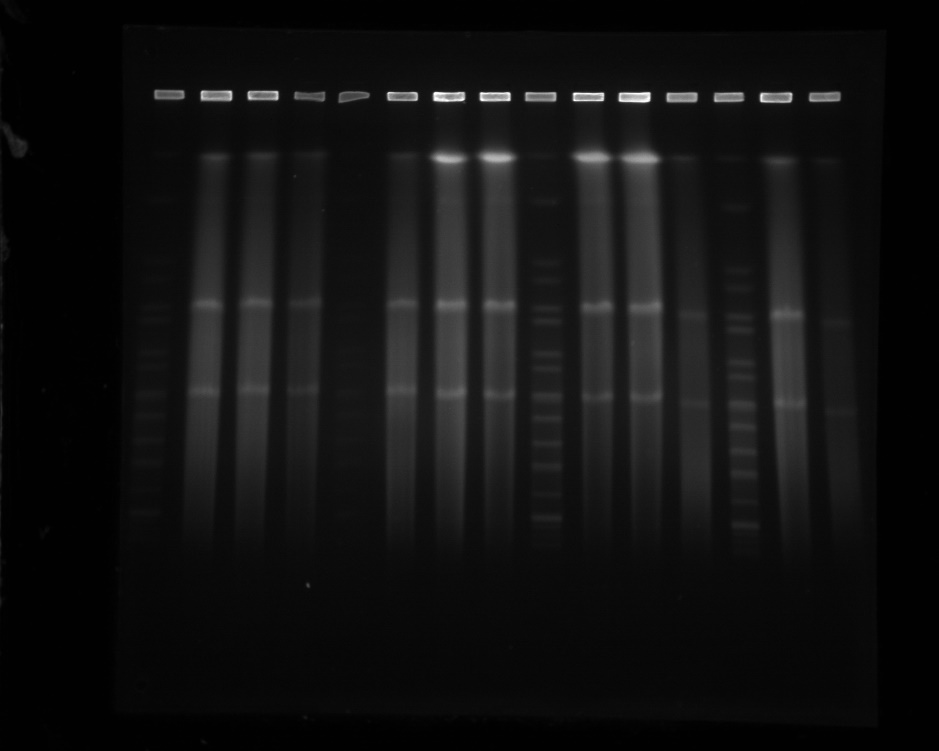


1. The original, uncropped blot of the *bla*_IMP-4_ gene (in the middle of the photo)


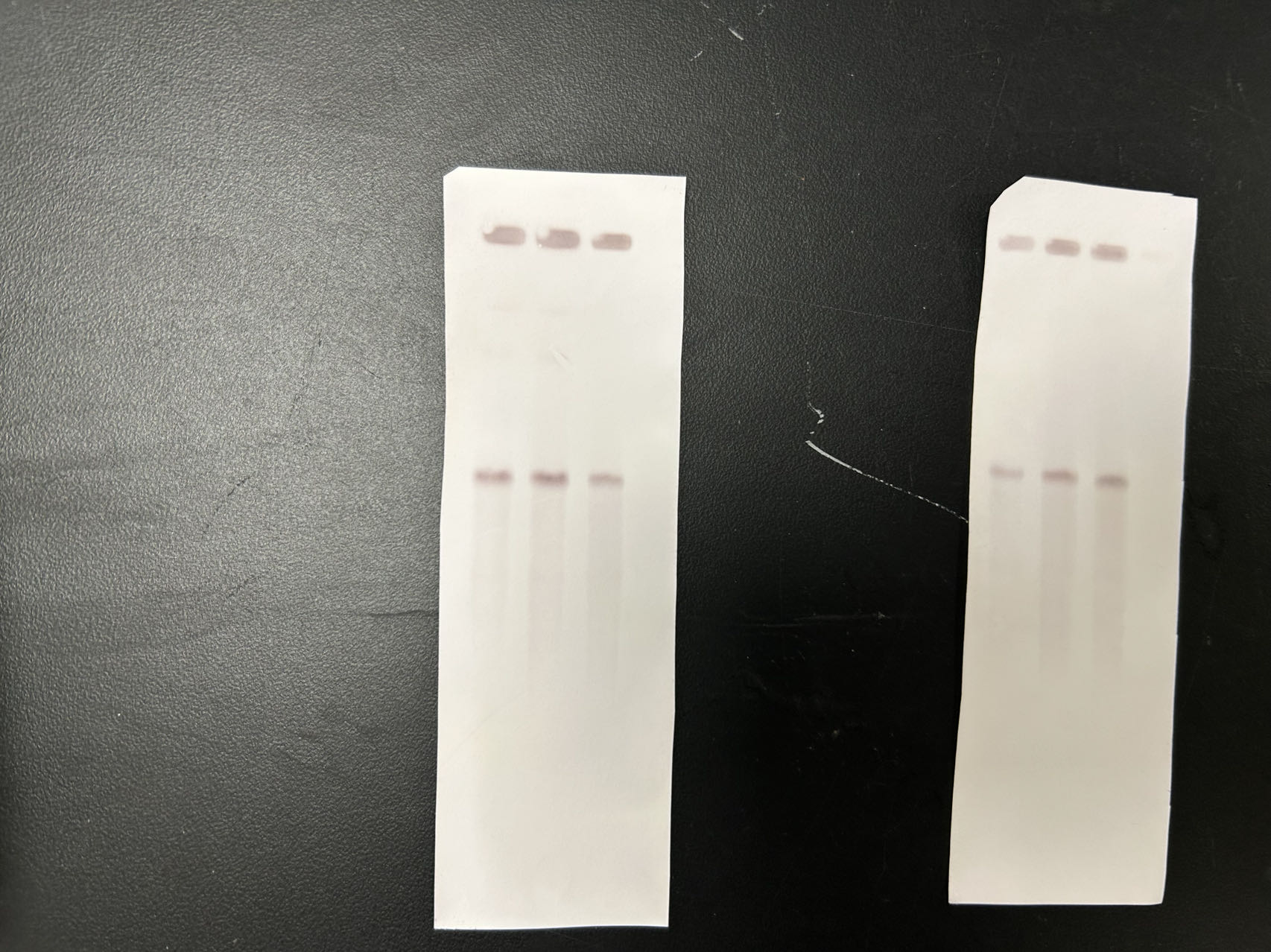


1. 3. The original, uncropped blot of the *bla*_SFO-1_ gene (in the middle of the photo)


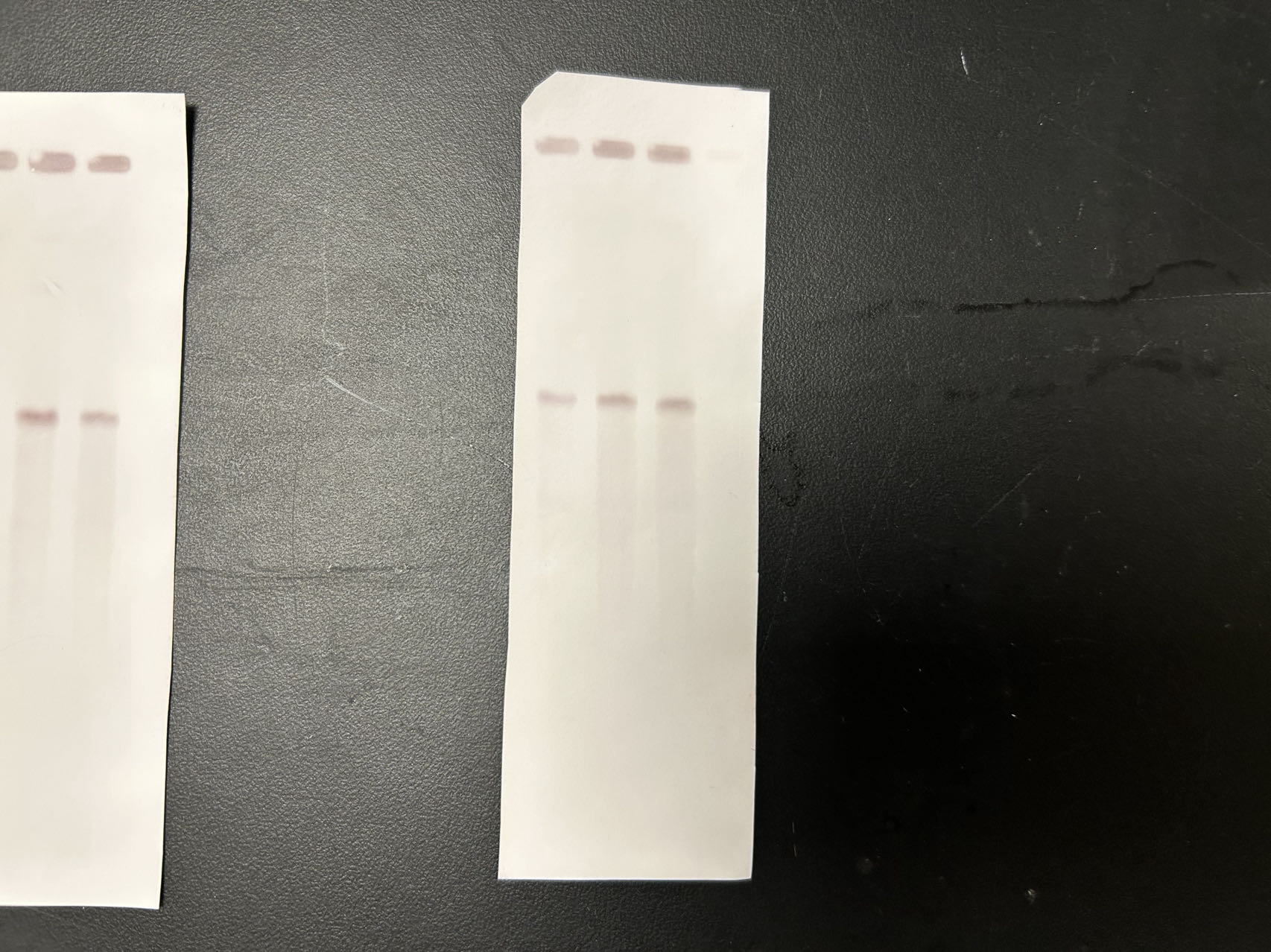

Supplement: Supplementary file 1 — Supplementary Material 1 [file 12941_2024_680_MOESM1_ESM.docx]
