# Supplementary material for: Coexistence of blaIMP−4 and blaSFO−1 in an IncHI5B plasmid harbored by tigecycline-non-susceptible Klebsiella variicola strain
Source: Ann Clin Microbiol Antimicrob. 2024 Mar 6;23:24. doi: 10.1186/s12941-024-00680-9 (PMC10918965; doi:10.1186/s12941-024-00680-9)
Supplement: Supplementary file 2 — Supplementary Material 2 [file 12941_2024_680_MOESM2_ESM.docx]

**Table S1** primers for PCR and the probe of Southern blotting

| Primers | Sequence |
| --- | --- |
| *imp-4*-F | GTAGCAAACTTCAATTGCCC |
| imp-4-R | AACAACCACCGAATAATATT |
| *sfo-1*-F | GTGCAGGCTCGAGCTCTTTT |
| *sfo-1*-R | AATGGCAGTATTTAAAGTAG |

**Table S2** Genomic features of *K. variicola* 4253

| Feature | Chromosome | p4253-imp | p4253-2 | p4253-3 |
| --- | --- | --- | --- | --- |
| Size(bp) | 5,533,843 | 334,271bp | 175,117bp | 4,655bp |
| GC content(%) | 57.3 | 48.7 | 51.5 | 42.8 |
| Inc group | NA | IncHI5B | IncFIB(K) | Col440I |
| No. of protein-coding sequences | 2,651 | 318 | 151 | 7 |
| No. of tRNA genes | 171 | 0 | 0 | 1 |
| No. of rRNA genes | 61 | 0 | 0 | 0 |
| Accession numbers | CP135068 | CP135069 | CP135070 | CP135071 |

NA, not applicable

**Table S3** Virulence genes of *K. variicola* 4253

| Genome | Virulence factors | Coding genes |
| --- | --- | --- |
| Chromosome | Adherence  Fimbrial adherence determinants | type 3 fimbriae (*mrkABCDFHIJ*), type 1 fimbriae (*fimABCDEFGHIK*)  *stcB, stcC* |
|  | Efflux pump | *acrA, acrB* |
|  | Iron uptake | aerobactin *(iutA),* ent siderophore*(entABCDEFS, fepABCDG, fes),* salmochelin *(iroE)* |
| p4253-imp  p4253-2  p4253-3 | Magnesium uptake  Regulation  Secretion system  None  Secretion system  None | *mgtB*  *rcsA, rcsB*  T6SS-I *(tssJGFM),* T6SS-II *(clpV), T6SS-III (dotU, icmF, impJGHFA, ompA, sciN,vgrG)*  None  T6SS-I (*tssH*)  None |

| Plasmid | Species | Size(bp) | Year of isolation | Source | Country | Genbank  accession no |
| --- | --- | --- | --- | --- | --- | --- |
| pNDM-IMP-1 | *K. variicola* | 347,317 | 2018 | Clinical | China | CP050681.1 |
| pKOX7525_1 | K. michiganensis | 397,447 | 2020 | Clinical | China | CP065475.1 |
| pWH11 | K. pneumoniae | 325,030 | NA | Clinical | China | ON882017.1 |
| pIMP4-KP294 | K. pneumoniae | 349,403 | 2020 | Clinical | China | CP083446.1 |
| pKP1814-1 | K. pneumoniae | 299,858 | NA | Clinical | China | KX839207.1 |
| pA | K. quasipneumoniae | 311,723 | 2018 | Clinical | China | CP068445.1 |
| p2019SCSN059_tmexCD_333k | K. quasipneumoniae | 333,095 | 2019 | Clinical | China | ON169978.1 |
| pFK2020ZBJ35_tmexCD_325k | K. variicola | 325,393 | 2019 | Clinical | China | ON169979.1 |

**Table S4** Information about the related plasmids
